# Mode Sensitivity Exploration of Silica–Titania Waveguide for Refractive Index Sensing Applications

**DOI:** 10.3390/s21227452

**Published:** 2021-11-09

**Authors:** Muhammad A. Butt, Andrzej Kaźmierczak, Cuma Tyszkiewicz, Paweł Karasiński, Ryszard Piramidowicz

**Affiliations:** 1 Warsaw University of Technology, Institute of Microelectronics and Optoelectronics, Koszykowa 75, 00-662 Warszawa, Poland; andrzej.kazmierczak@pw.edu.pl (A.K.); ryszard.piramidowicz@pw.edu.pl (R.P.); 2 Silesian University of Technology, Department of Optoelectronics, Krzywoustego 2, 44-100 Gliwice, Poland; cuma.tyszkiewicz@polsl.pl (C.T.); pawel.karasinski@polsl.pl (P.K.)

**Keywords:** silica–titania integrated waveguides, optical ring resonator, refractive index sensor, sol–gel film deposition, waveguide mode sensitivity

## Abstract

In this paper, a novel and cost-effective photonic platform based on silica–titania material is discussed. The silica–titania thin films were grown utilizing the sol–gel dip-coating method and characterized with the help of the prism-insertion technique. Afterwards, the mode sensitivity analysis of the silica–titania ridge waveguide is investigated via the finite element method. Silica–titania waveguide systems are highly attractive due to their ease of development, low fabrication cost, low propagation losses and operation in both visible and near-infrared wavelength ranges. Finally, a ring resonator (RR) sensor device was modelled for refractive index sensing applications, offering a sensitivity of 230 nm/RIU, a figure of merit (*FOM*) of 418.2 RIU^−1^, and *Q-factor* of 2247.5 at the improved geometric parameters. We believe that the abovementioned integrated photonics platform is highly suitable for high-performance and economically reasonable optical sensing devices.

## 1. Introduction

Silica, titania, and silica–titania compounds obtained by the sol–gel process have been extensively investigated because of their potential optical functions [1,2]. They have been manipulated to build planar (1D) and ridge (2D) waveguides (hereafter represented as WGs) with excellent optical properties and negligible optical transmission losses for photonic integrated circuits (PICs) [3,4,5]. The sol–gel methodology is uncomplicated and cheaper [6]. It is capable of coating large areas and does not need high-temperature processing. It permits the development of high-quality thin films with excellent thermal and mechanical stability [7,8].

A suitable refractive index contrast between the WG layer and substrate is essential for WG operation. Light propagates in the high-index thin film, where it is confined by the mechanism of total internal reflection (TIR) [9]. Adjusting the set-off precursors and solvents, as well as the stoichiometric ratio between the silica and titania compounds and the thermal treatment temperature, will regulate the refractive index and thickness of the thin films formed by the sol–gel method. These WGs can be doped with rare-earth elements, laser dyes, and other organic compounds, enabling silica and titania materials created by the sol–gel method to be used in optical amplifiers, laser-active media, and sensing applications [10,11]. The sol–gel method is exceedingly effective and does not require expensive high-tech apparatus. By using the sol–gel method, the refractive index of the WG films can be controlled, and the optical losses suffered by these WGs are comparable to WGs acquired from the chemical vapor deposition (CVD)/low-pressure chemical vapor deposition (LPCVD) method [12,13]. In our previous studies [13,14,15], rib WGs and directional couplers (DCs) were fabricated by using traditional optical photolithography and wet chemical etching in SiO_2_:TiO_2_ WG layers.

In this paper, we report advances in the development of a novel WG material platform based on SiO_2_:TiO_2_ material for applications in integrated photonic devices. Moreover, numerical analysis of the mode sensitivity of the SiO_2_:TiO_2_ ridge WG is presented. The work is concluded with a numerical analysis of a simple optical ring resonator (RR)-based sensor device to be fabricated using the presented technology. The conducted simulations of the proposed optical RR sensor structure have proven its potentially high sensitivity comparable with the sensitivity offered by the leading configurations of competitive photonic sensors. For example, when comparing our sensor with a plasmonic sensor based on Au/Si nanorods [16], one can state that the sensitivity offered by our device is of the same order of magnitude (230 nm/RIU in the case of SiO_2_:TiO_2_ RR vs. 340 nm/RIU). However, our sensor offers a much better quality factor (2247.5 vs. 10.09); consequently, it should be much easier to identify a central wavelength of the resonance peak and correctly identify the resonance peak shift. Considering the potentially much easier fabrication technology (with the use of direct nanoimprint) and cascading devices (with a serial connection of several RR), the proposed design of the sensor device is a very promising candidate for future implementation in multiparametric lab-on-a-chip sensors.

## 2. SiO_2_:TiO_2_ Sol–Gel-Derived WG Films

**Figure 1:**
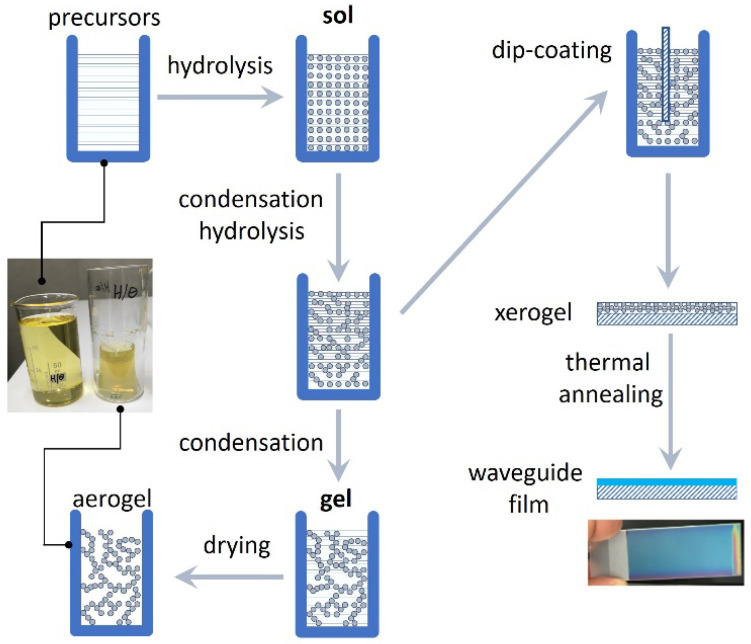
Schematic representation of the SiO_2_:TiO_2_ WG film deposition process.

In our research, we consider the use of low-propagation-loss SiO_2_:TiO_2_ WG layers that are fabricated using sol–gel-derived deposition method with a dip-coating technique. This technology has been mastered at the Silesian University of Technology [6]. The fabrication procedure is depicted schematically in Figure 1, as quite a straightforward method and does not require an expensive setup.

In this process, tetraethyl ortotitanate (TET) is used as a titanium dioxide (TiO_2_) precursor, and tetraethyl ortosilicate (TEOS) is used as silicon dioxide (SiO_2_). The two precursors do not dilute in water; therefore, the ethyl alcohol (C_2_H_5_OH) is used as the homogenizing factor. Hydrolysis and condensation reactions are catalyzed with the use of hydrochloric acid.

A proper rectangular glass substrate is dip-coated in fabricated sol and subsequently annealed by heating it up to a temperature of 400 °C for 30 min.

This fabrication procedure has several fundamental advantages, including:

- Simplicity and low-cost fabrication because no expensive equipment, such as a PECVD system, is necessary;
- Precise control of WG thickness with proper adjustment of the substrate withdrawal speed;
- Precise control of refractive index with proper adjustment of the stoichiometric ratio between precursor components;
- Low propagation loss thanks to the high smoothness of fabricated WG films;
- Possibility of etch-less fabrication with the implementation of direct nano-imprint lithography (NIL) on non-hardened WG films;
- Scalability with an increment of the number of substrates being dip-coated at the same time.

The first four of the above-mentioned advantages have been demonstrated in our previous research [6]. The possibility of using the NIL technique was partially demonstrated with the development of shallowly patterned vertical grating coupler sensors [17]. The scalability will be investigated in our forthcoming research after the successful development of a fully efficient WG film fabrication and patterning technique when the technology will reach the industrial maturity standard.

**Figure 2:**
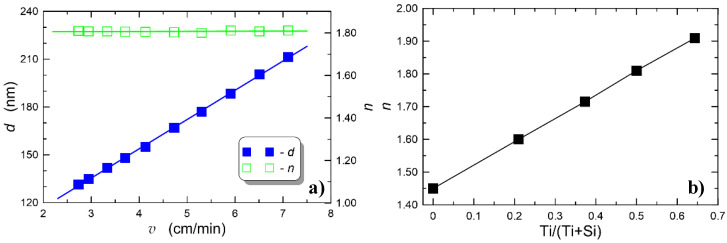
Dependence of WG film thickness and refractive index on withdrawal speed of the substrate from the sol (**a**), and the dependence of refractive index on the stoichiometric ratio between Si and Ti components in the sol (**b**).

The possibility of precisely controlling the SiO_2_:TiO_2_ WG film thickness and the refractive index was investigated by the procurement and characterization of a series of films fabricated with different SiO_2_:TiO_2_ precursor ratios and different substrate withdrawal speeds from the sol. Fabricated WG films were investigated using the optical ellipsometry technique; the obtained results are shown in Figure 2.

As one can observe in Figure 2a, the thickness of the WG film depends linearly on the withdrawal speed from sol, whereas the refractive index depends on *v* only to a minor extent. The concentration of titanium in a sol, governed by the relative concentration of respective precursors of silica and titania, has a considerable impact on the refractive index of composite SiO_x_:TiO_y_ WG films. The effect of titanium concentration on the refractive index of such WG films is shown in Figure 2b. Square markers corresponding to refractive index values were obtained experimentally. Characteristics presented in Figure 2a were obtained for a composition characterized by the ratio of Ti:Si = 1:1.

**Figure 3:**
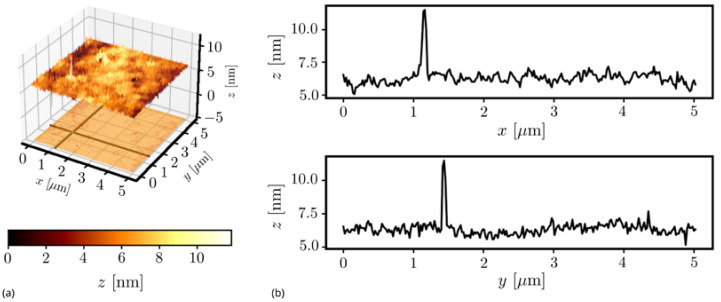
An AFM image (**a**) and roughness profiles (**b**) of the top surface of the SiO_2_:TiO_2_ film deposited on a soda–lime glass substrate.

The surface topology of SiO_2_–TiO_2_ films fabricated on soda–lime glass substrates was investigated using atomic force microscopy (AFM). An exemplary AFM image (left) and two-axis top-surface line scans (right) are shown in Figure 3.

**Figure 4:**
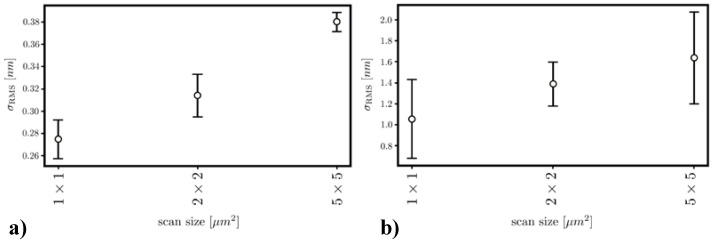
Variation of the root mean square surface roughness and its maximum deviation from the average concerning scan field dimensions for (**a**) SiO_2_:TiO_2_ film deposited on a BK7 glass substrate and (**b**) a bare substrate.

The selected sample was measured in nine different spots using the Ntegra Spectra system. Eight spots were located on the circumference of a square with dimensions of 10 × 10 mm^2^. The last, the ninth, was located in the center of that square. There were registered scans of fields having dimensions of 5 × 5 µm^2^, 2 × 2 µm^2^ and 1 × 1 µm^2^, three per each size. This enabled determining the dependence of root mean square surface roughness σ_RMS_ and its maximum deviation from the average on scan dimensions, as presented in Figure 4.

**Figure 5:**
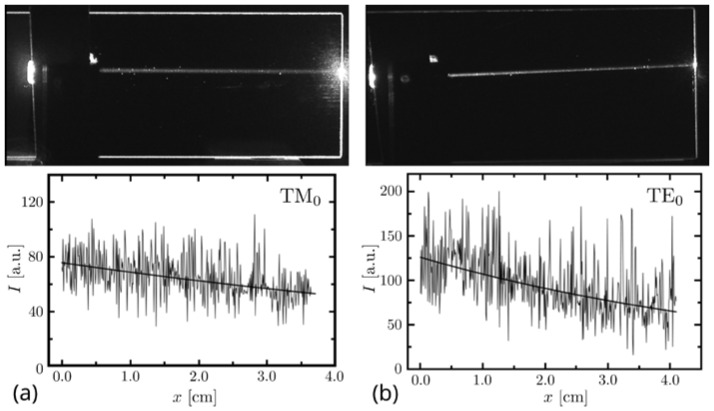
Demonstration of the light propagation in SiO_2_:TiO_2_ WG films. Light intensity *I* is given in arbitrary units. A scattering streak of light (top) and its intensity profile (bottom)is shown for the TM (**a**) and TE (**b**) polarization.

The surface roughness increases with the length of the probed area, because on larger areas, it is more likely to encounter sharp peaks such as that present in Figure 4a and whose center coordinates are x = 1.16 µm and y = 1.43 µm. Nevertheless, the roughness of SiO_2_–TiO_2_ films is very low. It is approximately four times lower than the surface roughness of the BK7 glass substrate (Figure 4b). Additionally, the operation of fabricated WG films was verified by inserting light into the WG slab (using the prism-insertion technique) and observing the stray light propagating in the WG. In Figure 5, two SiO_2_:TiO_2_ WG films excited with light of wavelength *λ* = 677 nm are shown. Each picture shows a streak of light scattered on WG film interfaces accompanied by the distribution of light intensity *I* recorded along with the streak. Intensity is expressed in arbitrary units falling within the interval from *I* = 0 to *I* = 255, because light streaks were registered using a monochromatic Panasonic WV-BL730 camera. Its CCD sensor allows recording images with 8-bit depth.

The experiment demonstrated in Figure 5 clearly shows that the fabricated WG films have very good properties in terms of light propagation. Based on the demonstrated intensity profiles, the value of the propagation loss for these WG films has been estimated to be α = (0.34 ± 0.03) dB/cm for TM_0_ mode and α = (0.71 ± 0.08) dB/cm for TE_0_ mode.

With accomplished experimental investigation, it was possible to confirm that the developed WG film fabrication technology allows for full control of the film thickness and refractive index of procured WG films. Furthermore, the fabricated films have very smooth surfaces, resulting in low propagation loss. It is therefore quite fair to state that the developed films are good candidates for further use in the construction of highly integrated sensor structures, the designs of which will be discussed hereafter in forthcoming sections of the paper.

The fabrication procedure for more complex WG structures (including optical RRs) using developed SiO_2_:TiO_2_ WG films are currently being developed in a twofold manner. In a more conventional procedure, the WG film is deposited using a dip-coating technique from the sol and subsequently thermally annealed as shown in Figure 1. Furthermore, the electron beam resist is deposited on the WG film, and e-beam lithography is used to pattern the resist. The WG film will subsequently be dry etched to replicate the pattern from the resist.

In a more innovative (and potentially with less fabrication effort required) procedure, the dip-coating step will be followed by direct nanoimprinting of the RR pattern on non-hardened WG film and subsequent thermal hardening of patterned WG structures. Both solutions are currently under development.

## 3. Effective Refractive Index Analysis

**Figure 6:**
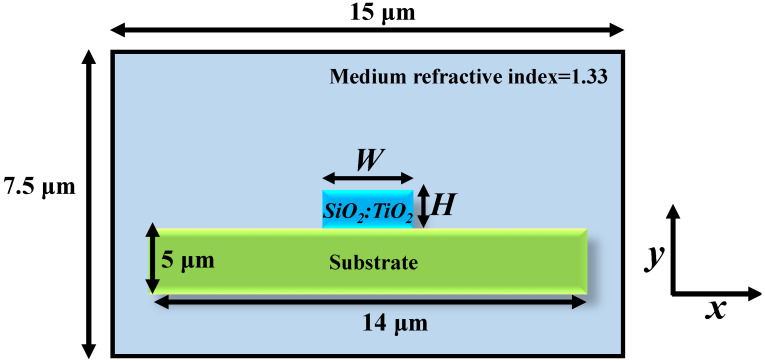
SiO_2_:TiO_2_ ridge WG scheme.

Figure 6 presents a schematic representation of a WG structure that will be investigated hereafter. It is a ridge WG composed of a SiO_2_:TiO_2_ core deposited on a standard glass substrate. The width and height of the WG core are denoted as *W* and *H*, respectively. The refractive indexes of the core and substrate were fixed at 1.8 (see Figure 2) and 1.5135 [18], respectively, which corresponded to the refractive index of fabricated WG films. The WG was placed in an aqueous medium of refractive index = 1.33. The numerical analysis was conducted via a 2D finite element method (2D-FEM) in COMSOL Multiphysics software. The entire device structure was divided into triangular elements with a predefined “Extremely fine” mesh grid size. The effective refractive index of the propagating mode was calculated for a WG of different heights and utilized to solve the problem. Scattering boundary conditions (SBCs) were applied on the outer boundary of the WG geometry to make the boundary transparent for the scattered electromagnetic waves. The sensing capability of the abovementioned platform was analyzed by modelling the RR structure for refractive index sensing applications.

First, the real part of the effective refractive index (*n_eff_*) of the propagating mode was calculated for different geometric parameters of the WG core. As shown in the previous section, in our lab, we can deposit a smooth, thin layer of SiO_2_:TiO_2_ on glass with the help of the sol–gel dip-coating method [6,19]. Furthermore, the WG film thickness can be precisely adjusted with the properly selected withdrawal speed of the substrate from sol. In [20], a linear dependence between the withdrawal speed of the sample and the thickness of the deposited SiO_2_:TiO_2_ film has been shown, with the film thickness ranging from 200 nm to 350 nm. Recently, with further technological development, we have been able to demonstrate WG films with thicknesses reaching 500 nm. Consequently, the mode behavior has been analyzed at different core dimensions.

**Figure 7:**
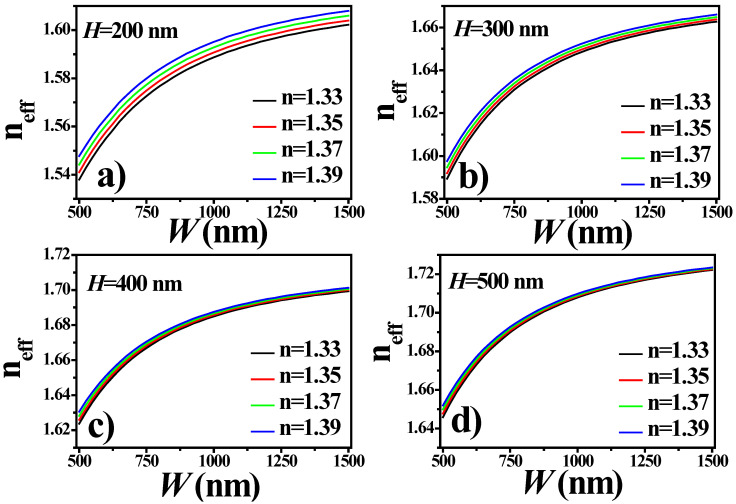
Re(*n_eff_*) versus W for the H of (**a**) 200 nm, (**b**) 300 nm, (**c**) 400 nm and (**d**) 500 nm. Please note that only the TE-fundamental mode was considered.

Figure 7a–d present the Re(*n_eff_*) of the TE-mode at 677 nm. The *n_eff_* of the mode increases as *H* and *W* of the WG core increases, which leads to the strong confinement of light in the core. This attribute is beneficial for designing optical interconnects where low transmission losses are desirable. However, for sensing applications, slightly leaky modes are favorable because they can provide enhanced sensitivity due to the strong light–matter interaction [21]. The mode propagating in the WG of *H* = 200 nm is highly sensitive to the ambient medium as compared to the mode propagating in the WG core of *H* = 500 nm. This is because a large portion of the evanescent field is propagating on the surface of the WG core. When the ambient refractive index increases from 1.33 to 1.39, a shift in the n_eff_ is observed, with the rate of shift dependent on the *H* of the WG core.

**Figure 8:**
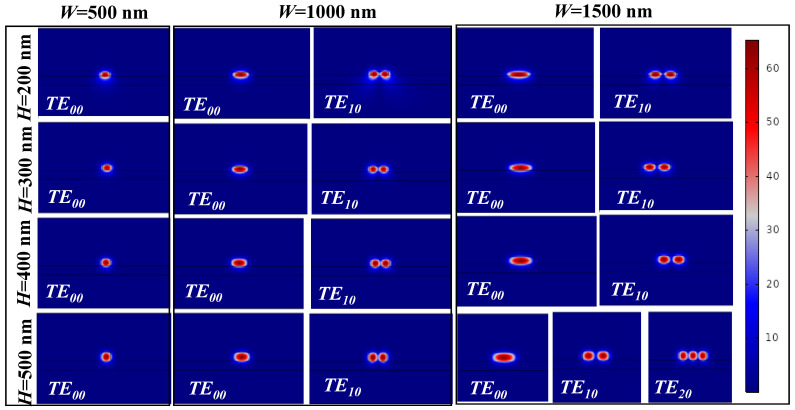
TE-mode distribution in ridge WG with different geometric parameters at an operational wavelength of 677 nm.

For optical communication or sensing applications, single-mode WG operation is appropriate. Therefore, *n_eff_* calculation for WG geometry is an initial step to designing WGs of any type. In Figure 8, the normalized E-field distribution of the TE-polarized mode is plotted. For *W* = 500 nm, the WG supports a fundamental mode for *H* = 200 nm, 300 nm, 400 nm and 500 nm. However, as *W* approaches 1000 nm and beyond, the WG geometry starts supporting higher-order modes (TE_10_ and T_20_). Therefore, special attention should be given while designing optical devices where single-mode operation is desirable.

## 4. Mode Sensitivity Analysis

**Figure 9:**
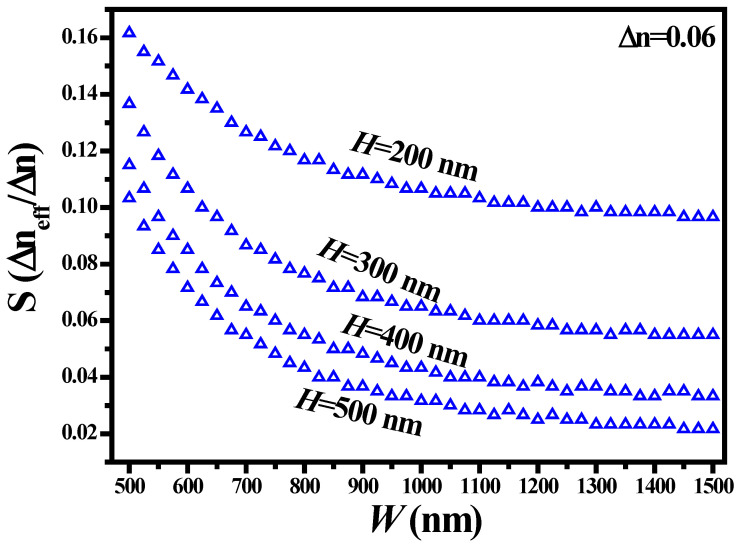
Mode sensitivity analysis of a ridge WG.

The mode sensitivity analysis of a WG structure is vital. Based on this information, suitable WG dimensions are selected for sensing applications [22,23]. It can be determined by utilizing the following expression:

(1) $$S_{mode}=\frac{\Delta n_{eff}}{\Delta n},$$

where Δ*n_eff_* and Δ*n* are the change in the effective refractive index and change in ambient refractive index, respectively. From Figure 9, it can be seen that the propagating mode is highly responsive to the ambient medium at small values of *H* and *W*. For instance, a mode sensitivity of 0.162 Δ*n_eff_*/Δ*n* was acquired for the WG geometry of *W* = 500 nm and *H* = 200 nm for Δ*n* = 0.06. On the other hand, the sensitivity of the WG reduced to 0.097 Δ*n_eff_*/Δ*n* at *W* = 1500 nm and *H* = 200 nm. Further reductions in sensitivity were noticeable as *H* increased to 500 nm. However, the highly sensitive devices produced a drawback of high propagation loss of the mode. Therefore, there is always a compromise between high sensitivity and high optical losses.

## 5. Ring Resonator Sensor Based on a Silica–Titania Platform

Furthermore, an RR device was modelled to numerically investigate the refractive index sensing capabilities of the SiO_2_:TiO_2_ optical WG. Fabrication of the proposed RR structure is currently in progress. There are three vital parameters, sensitivity (*S*), figure of merit (*FOM*) and *Q-factor*, which should be well-thought-out while designing the sensing devices [24,25]. The sensitivity of the RR is calculated by using the following expression [26]:

(2) $$S=\frac{\Delta \lambda_{res}}{\Delta n},$$

where Δ*λ_res_* and Δ*n* are the change in resonance wavelength and ambient refractive index, respectively. *FOM* is the ratio between the sensitivity and full width at half maximum (FWHM) of the resonance dip, which is expressed as:

(3) $$FOM=\frac{S}{FWHM},$$

Integrated resonators with high *Q-factors* are principally required for a wide range of applications, for instance, narrow-bandwidth filters, high-efficiency non-linear optic devices, high-performance lasers and high-sensitivity sensors [27,28,29,30]. The *Q-factor* of the proposed device can be calculated as:

(4) $$Q-factor=\frac{\lambda_{res}}{FWHM},$$

**Figure 10:**
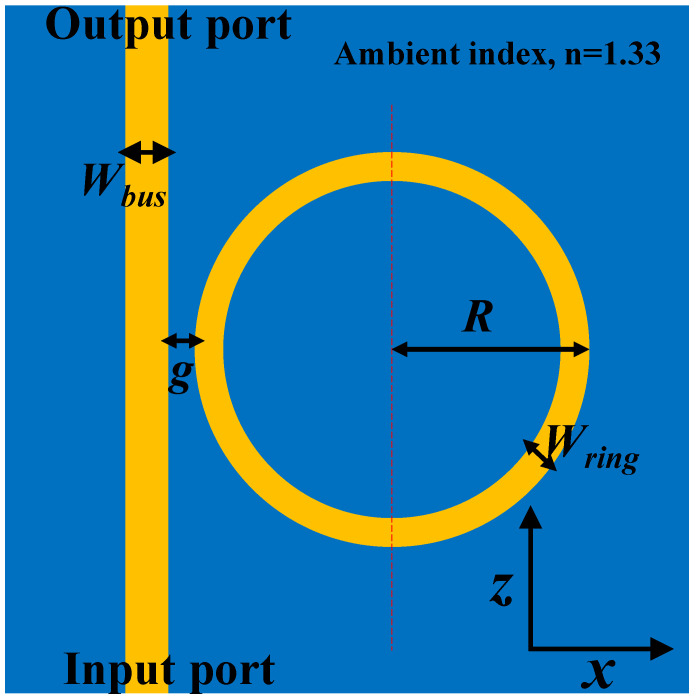
Schematic representation of an RR based on SiO_2_:TiO_2_ optical WG.

The RRs are highly responsive to changes in the ambient medium, which result in the redshift of the resonance wavelength [31]. The amount of shift in the resonance wavelength is dependent on the geometric parameters of the device. The schematic of the RR is shown in Figure 10, where *W_bus_* and *W_ring_* represent the width of the bus WG and ring WG, respectively.

**Figure 11:**
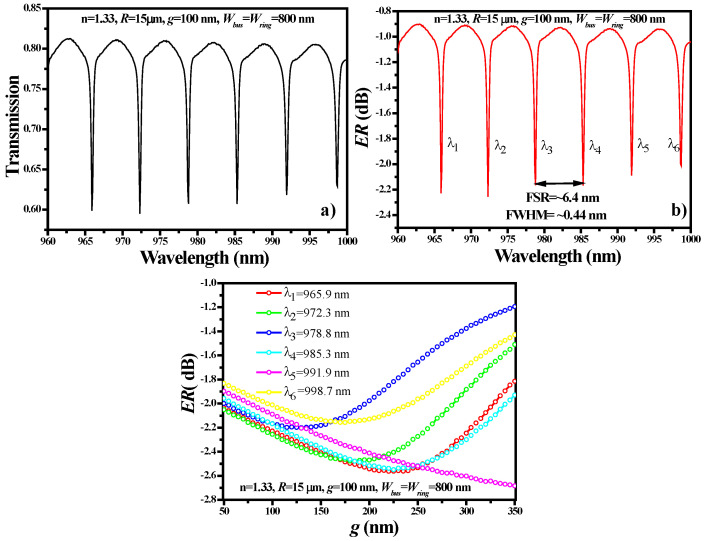
Spectral characteristics of the RR structure: (**a**) transmission spectrum, (**b**) FSR versus ER, (**c**) optimization of *g*. Geometric parameters such as *R* = 15 μm, *g* = 100 nm, *W_bus_* = *W_ring_* = 800 nm and *n* = 1.33 are used.

The radius of the ring (denoted as *R*) was chosen to be 15 μm based on the computational limit of our computer. The coupling distance between the bus WG and the ring is denoted as *g*. The ambient medium was filled with a dielectric material of *n* = 1.33. From a practical point of view, the height (*H*) of the device design was fixed at 200 nm, which could be obtained in a single dip-coating process, and the *n_eff_* values of the Si WG were obtained from Figure 7. A TE-polarized plane wave was coupled at the input port of the bus WG, and the output power was collected at the other end. To determine the free spectral range (FSR) of the RR structure, the extinction ratio (*ER*) was calculated by using the following expression:

(5) $$ER\;\left(\mathrm{dB}\right)=10\times log\left(\frac{P_{out}}{P_{in}}\right),\;$$

where *P_out_* and *P_in_* are the power at the input and output port of the bus WG, respectively. FSR was calculated in the near-IR wavelength range of 960 nm to 1000 nm by utilizing a “parametric sweep function” with a step size of 0.05 nm. This wavelength range is suitable for the ring structure of *H* = 200 nm; otherwise, in the high-wavelength range, the WG becomes leaky, and light penetrates the substrate. For Figure 11a–c, the geometric parameters of the ring structure used for the analysis are as follows: *R* = 15 μm, *g* = 100 nm, *W_bus_ = W_ring_* = 800 nm and *n* = 1.33. The transmission spectrum of the RR is plotted in Figure 11a. It is well noting that six resonance dips (*λ*_dip_) were obtained with an FSR of ~6.4 nm and the FWHM of *λ*_dip_ was ~0.44 nm, as shown in Figure 11b.

For each *λ*_dip_, the coupling efficiency (CE) of the light from the bus WG to ring structure was determined as shown in Figure 11c. CE can be determined by the following two methods: (1) integrating the ring structure to calculate the energy stored in it at resonance wavelength; and (2) calculating the *ER* of the bus WG. Both methods are feasible; however, we have selected the second method and obtained the *ER* for *g* in the range of 50 nm to 350 nm. The maximum *ER* of ~2.5–2.6 dB was obtained for *g* = 250 nm to 300 nm. In the sensing analysis section, we fixed *g* = 225 nm, which is one of the optimized coupling gaps to obtain high *ER*.

**Figure 12:**
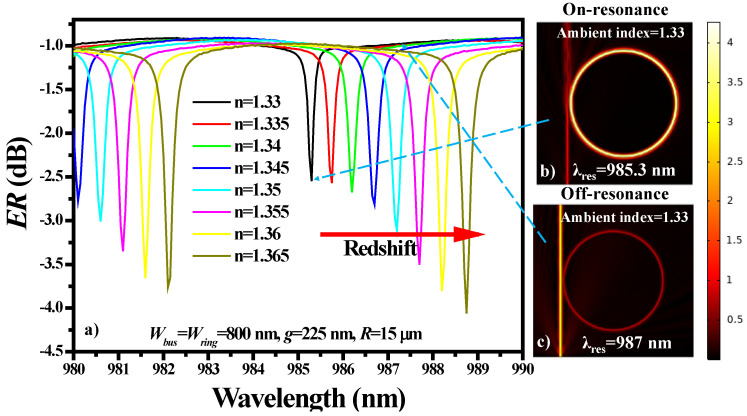
(**a**) Transmission spectrum of the RR structure in the presence of different ambient refractive indices. Normalized E-field distribution in the RR structure in the (**b**) on-resonance state and (**c**) off-resonance state. The geometric parameters of the structure are as follows: *R* = 15 μm, *g* = 225 nm, and *W_bus_* = *W_ring_* = 800 nm.

The sensing capabilities of the proposed sensor design were evaluated by varying the ambient refractive index from 1.33 to 1.365 with a step size of 0.005. The transmission spectrum (dB) of the RR is plotted for a wavelength range of 980 nm to 990 nm where a *λ*_4_ dip is present. The *λ*_dip_ performed a redshift as the refractive index of the ambient medium increased, as shown in Figure 12a. For this analysis, *W_bus_ = W_ring_* = 800 nm was fixed, which provides strong confinement of light in the ring structure which results in a weak evanescent field. Consequently, light–matter interaction is low, resulting in *S* = ~90 nm/RIU, *FOM* = ~204.5 RIU^−1^ and *Q-factor* = 2239. Normalized E-field distributions at *λ* = 985.3 nm and *λ* = 987 nm are plotted in Figure 12b,c, which represent the on-resonance state and off-resonance state, respectively.

**Figure 13:**
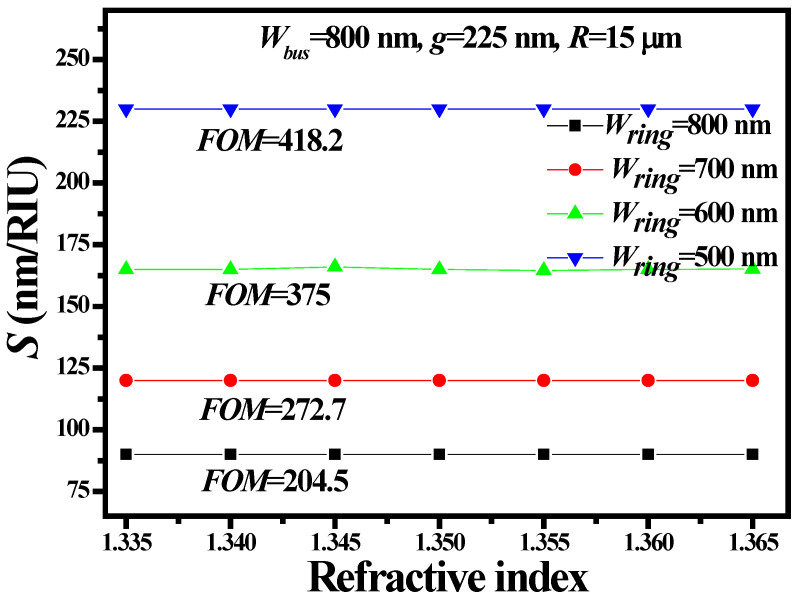
Evaluation of *S* and *FOM* based on *W_ring_*. The remaining geometric parameters such as *W_bus_, R* and *g* were fixed at 800 nm, 15 μm and 225 nm, respectively.

As mentioned earlier, sensitivity can be enhanced by increasing the light–matter interaction, i.e., increasing the evanescent field ratio (*EFR*). *EFR* is defined as the ratio of E-field power in the upper cladding and the total power of the WG mode [32,33]. Higher *EFR* can be obtained by decreasing the width of the ring WG (*W_ring_*). The device performance is calculated by reducing *W_ring_* from 800 nm to 500 nm with a step size of 100 nm. The *λ*_res_ at *W_ring_* = 500 nm is 988.9 nm with an FWHM of 0.44 nm. The remaining parameters such as *W_bus_, g* and *R* were maintained at 800 nm, 225 nm and 15 μm, respectively. The *S*, *FOM* and *Q-factor* of the proposed device were enhanced to ~230 nm/RIU and ~418.2 RIU^−1^ and 2247.5, respectively, by reducing *W_ring_* = 500 nm, as shown in Figure 13. Table 1 summarizes the device performance at different values of *W_ring_.*

The results presented in this work are highly attractive and comparable to the well-known silicon photonics and optical fiber-based sensors. For instance, a semiconductor nanowire refractive index sensor for biosensing application was verified, offering *S* = 235 nm/RIU [34]. A long-period fiber grating sensor has been proposed for refractive index sensing applications [35]. The obtained sensitivity value is comparable with silicon nitride slot WG RRs sensors, as shown by Carlborg et al., with an experimental sensitivity of 246 nm/RIU with a much more complex RR layout [36]. However, by adjusting the WG thickness, *S*_bulk_ = 270 nm/RIU was demonstrated [37].

## 6. Conclusions

In conclusion, a highly attractive WG platform based on SiO_2_:TiO_2_ is presented, which can be easily developed at low costs. The wide transparency range of the material makes it an ideal candidate to be employed in the visible and near-IR wavelength range. A mode sensitivity analysis of the WG demonstrates the practical applicability of this system in the sensing field. In the end, an RR device has been proposed for refractive index sensing applications and offers sensitivity in the range of 90 nm/RIU to 230 nm/RIU based on the geometric parameters of the WG. We believe that this SiO_2_:TiO_2_ platform can find several applications in the implementation of active and passive optical components.

## Figures and Tables

**Table 1:** RR performance for different values of *W_ring_*.

*W_ring_* (nm)	*S* (nm/RIU)	*FOM* (RIU^−1^)	*Q-Factor*
500	~230	~418.2	2247.5
600	~165	~375	2240
700	~120	~272.7	2227.5
800	~90	~204.5	2239
